# Unraveling the controversial effect of Covid-19 on college students’ performance

**DOI:** 10.1038/s41598-023-42814-7

**Published:** 2023-09-23

**Authors:** Luca Bonacini, Giovanni Gallo, Fabrizio Patriarca

**Affiliations:** 1 Department of Economics, University of Bologna, 40126 Bologna, Italy; 2 Department of Economics Marco Biagi, University of Modena and Reggio Emilia, 41121 Modena, Italy

**Keywords:** Health care economics, Social evolution, Epidemiology

## Abstract

We disentangle the channels through which Covid-19 has affected the performance of university students by setting up an econometric strategy to identify separately changes in both teaching and evaluation modes, and the short and long term effects of mobility restrictions. We exploit full and detailed information from the administrative archives of one among the first universities to be shut down since the virus spread from Wuhan. The results help solving the inconsistencies in the literature by providing evidence of a composite picture where negative effects such as those caused by the sudden shift to remote learning and by the exposure to mobility restrictions, overlap to opposite effects due to a change in evaluation methods and home confinement during the exam’s preparation. Such overlap of conflicting effects, weakening the signaling role of tertiary education, would add to the learning loss by further exacerbating future consequences on the “Covid” generation.

## Introduction

There is a wide and varied literature stressing how the pandemic crisis has harmed the accumulation of human capital. In this article we focus on tertiary education and in particular on students’ performance. While in the case of primary and secondary education the literature converges on the emergence of a consistent learning deficit^1^, in the case of tertiary education the picture is much more controversial. To untangle the knot it is important to consider the variety of channels through which the pandemic might have affected students’ outcomes. Indeed, together with channels that affected all the population, both directly on health and indirectly through containment and lockdown measures, in the case of college education there are specific channels related to the shutdown of in presence activities as the sudden shift to remote learning, the temporary return of students to their places of origin and the change in student assessment methods that also shifted to online mode. Each channel has had impacts on different aspects of students’ careers, with different intensities and even in opposite directions. In our opinion, this composite picture helps explaining the lack of uniqueness of the evidence provided by the related literature developed so far.

Separating the overall effects between different channels requires very detailed data as to implement satisfactory econometric strategies to go beyond the identification of the overall effect based on simple comparison of pre- and post-pandemic values. For this purpose, we use the administrative data of one among the first Universities directly involved in the spread of the virus outside China: the University of Modena and Reggio Emilia. We can track between 2018 and 2021 a total of about 38,000 students, who have taken about 400,000 exams, with high-level details on the characteristics of examinations, study paths, background of students and teachers fixed effects.

By exploiting this rich dataset, we build an econometric strategy based on difference-in-differences estimations^2^ to analyze the exams marks by distinguishing between the contrasting effects of the change in teaching and in assessment modes, and then consider separately the effects of exposure to lockdown measures.

On the one hand, while the transition to distance learning may have had a negative impact on learning, as confirmed by the literature on lower levels of education^3,4^, the need to change the assessment method may have had an opposite effect on measured performance. Indeed, since the shift to online exams made more difficult to avoid plagiarism or other misconduct^5^, this might have incentivized students to cheat. Furthermore, the exams mode itself (e.g. alone or in the classroom, with interviews or quizzes) may have affected students’ performance during the exams, and finally also teachers evaluation attitudes could have become less stringent. To solve the possible overlap of contrasting effects and correct for the possible divergent dynamics of actual and measured students’ performance, we exploit the pre-existence of courses where classes were already given, though partially, in remote mode even before the pandemic, although exams mode were the same as for the other courses. In this way we can build a difference-in-differences identification strategy exploiting the heterogeneity related to the fact that the extent shift of teaching mode has been different though the change in assessment mode has been the same.

On the other one hand, we use the information on the exam date as to take into account the effect of lockdown measures. This information allows us to build a proxy of exposure to restriction which is both time and space varying, by matching the data on the pattern of restrictions in Italian regions. Indeed, the prolonged closure of a university with a supra-regional students pool, located in an area with a relatively high cost of living, has led the majority of students to return to their homes. This led to (exogenous) different exposures to containment measures, since they had a predominantly regional character.

The results also give us a composite evidence that help us explain the puzzled results found in the literature about the effects of lockdown measures: while the overall exposure to containment measures appears to have a significant negative impact on students, being confined at home during the preparation of the exams turns out instead to have had a positive effect.

In the next sections, after a review of the related literature, we lay out a description of the case study and of the data used. Next, we present the econometric strategy and then discuss the results. Before concluding, in the final session we also perform some robustness checks.

## Tertiary education and the pandemic

While the socio-economic consequences of the Covid-19 have been already studied in deep from many points of views, papers focused on the impact of the pandemic on higher education are still few and provide contradictory results. We can split this branch of literature into two groups of studies: those using pupils’ surveys^6–8^ and those considering data on students’ actual outcomes^9–12^. Overall, the first ones find negative effects of the pandemic, while the second ones mostly agree on the contrary.

A pioneering contribution is provided by^6^, which surveyed approximately 1500 students at one of the largest public institutions in the United States. To our knowledge, their analysis is the first trying to get the impact of the pandemic on students’ outcomes. Results show large negative effects. Due to Covid-19, 13% of students have delayed graduation, 40% have lost a job, internship, or job offer, and 29% expect to earn less at age 35. Moreover, these effects have been highly heterogeneous: one quarter of students increased their study time by more than 4 weekly hours due to Covid-19, while another quarter decreased their study time by more than 5 h per week. This heterogeneity often followed existing socioeconomic divides. Lower-income students are 55% more likely than their higher-income peers to have delayed graduation due to Covid-19.

In the same spirit^8^, conducted an online survey on 3163 Queens College students during the summer 2020. She analyses the effect of the Covid-19 outbreak on current and expected outcomes through an estimation of individual-level subjective treatment effects. She finds that due to the pandemic, between 14 and 34% of students considered to drop-out, as they think to lose their financial assistance, or to postpone their graduation. The pandemic also deprived 39% of students of their jobs and reduced their earnings by 35%. Finally, her analysis also reveals that the effect of the pandemic on social insecurity has been different on the basis of the students’ well-being as it has been deeper for students with a federal Pell grant than their peers.

Hu et al.^7^ make a contribution to the analysis on students’ self-perception as they differentiate their analysis to the previous ones asking about students’ conditions two years later since the outbreak of the pandemic, in the period between January 17 to February 25, 2022. They surveyed 151 college students in Northern Michigan asking how much their learning quality is influenced by the Covid-19 and they conclude that respondents’ education was severely affected by the pandemic, averaging a score of 7.58 on a scale of 10. These results suggest that the negative impact of Covid-19 on students’ self-perception is not limited to the short run.

Contrasting results are provided instead by the second stream of literature as in^10–12^. Gonzalez et al.^10^ analyze the effects of Covid-19 confinement on the autonomous learning performance of students in higher education through a sort of randomized control experiment. Their study relies on a field experiment with 458 students at Universidad Autonoma de Madrid. The control group corresponds to academic years 2017/2018 and 2018/2019. The experimental group comprehends students from 2019/2020. The results show a significant positive effect of the Covid-19 confinement on students’ performance as they changed their learning strategies to a more continuous habit. Similar results hold in^11^. They estimate the effects of online education during the Covid-19 lockdown on student performance through a difference-in-differences approach using administrative data from Chinese Middle Schools. They consider three schools in the same county in Baise City before and after the Covid-19 onset. School A is the control group, as it did not provide any online educational support to its students. School B and C (treatment group) used an online platform. They point out a positive effect of online education by 0.22 of a standard deviation on student academic results. They also found that the results are homogeneous between rural and urban students.

Other contributions mainly focus on the heterogeneity of the effect across groups, but even in none of these we can find an evidence of a decrease in overall performance. Rodríguez-Planas^8^ uses an event study approach to compare the gap between low-income students and their peers in the same University. She concludes that lower-income students with a lower performance during the pre-pandemic period outperformed their higher-income peers thanks to the different use of the flexible grading policy based on their financial and academic needs. In contrast, in the absence of the flexible grading policy, lower-income top-performing students would have underperformed relative to their higher-income counterparts. Engelhardt et al.^13^ compare university students’ performance in the first semester affected by Covid-19 to that of the previous three ones. They do not find significant differences in performance across periods. These results are confirmed also for low-income, first-generation, and minority students. Castellanos-Serrano et al.^14^ focus on the academic consequences of the Covid-19 in gender inequalities by several education performances. They consider 7477 students enrolled in just one faculty from the 2016/2017 to 2020/2021 academic years. Using a basic pre-post identification strategy, they find heterogeneous effects of the pandemic by sex since women’s results worsened in comparison to those of the pre-covid-19 period to a greater extent than for men. Besides, all sex slightly improved their results over the pandemic period. Maldonado and De Witte^15^ consider the last year of primary schools in the Dutch-speaking Flemish region of Belgium. Using a 6-year panel, they perform a linear regression model with a pre-post Covid variable and find that, on average, students of the 2020 cohort experienced significant learning losses. Moreover, inequality within and across schools increased as a result of the Covid-19 crisis. Altindag et al.^16^ leverage data from 15,000 students enrolled in a U.S. public university to investigate the performance of students in in-person compared to online courses during the pandemic. Using a student fixed effects model, the authors find that students in in-person courses fared better than online students with respect to their grades, the propensity to withdraw from the course, and the likelihood of receiving a passing grade. Agostinelli et al.^17^ decompose the potential channels operating through the online learning, peers interactions, and the time spent with the parents. They conclude that each of these channels contribute to higher educational inequality during the pandemic.

All these studies target at the overall impact of Covid-19. Differently, Bird et al.^9^ focus on the specific impact of the pandemic-triggered shift to online education. To do that they use data on students attending Virginia’s community colleges and set up an econometric strategy partially similar to that of part of our analysis: they use a difference-in-differences strategy in which the treatment groups is composed by the students enrolled in an in-person course and the control group is composed by the students which the course was provided online also before the Covid-19 widespread. Differently to the present contribution, their primary outcome of interest is the course completion, namely a binary variable equal to one whether the student received any grade sufficient (A, B, C, D, P + , or P), zero otherwise. The authors find that the shift to the online modality led to a modest decrease in course completion between 3 and 6%. This reduction in course completion is primarily driven by a large increase in course withdrawals (37% or + 2.7 percentage points in absolute terms) and, more narrowly, by an increase in course failure (10.8% or + 1.3 percentage points).

It is thus worth to notice that by focusing on a specific channel of the impact of the Covid-19 period, results shows a different picture than the one offered by the aggregate evidence. Delving deeper in this direction, in this paper we will try to solve the apparent puzzle. Our basic hypothesis is that the coexistence of negative effects reported subjectively or detected in the analysis of specific channels, together with positive effects resulting from the analysis of the overall outcomes is mainly due to the coexistence of positive effects on reported performance due to a change in evaluation standards, and negative effects on actual performance.

## The case study

The case study is the University of Modena and Reggio Emilia. Unimore is a medium-sized Italian university, with a wide range of fields organized in 12 departments, ranked in the middle among Italian high education institutions, with a predominantly regional and national enrollment pool. As we will see in the econometric strategy session, this last characteristic together with the peculiarities of the relationship with pandemic events will be valuable for the purpose of the identification strategy we will use in this study. A final feature of the case study, that we will exploit in “Econometric strategy” section, is that a significant share of Unimore’s departments, before the pandemic, already offered degree programs where each single course provides mixed in-presence and remote classes.

At the same time, the university has recently undertaken a process of integrating all micro-data from administrative sources or interviews into a single database, Unimoredata, which enable us to analyze with a very high level of detail the performance trends of its students along the period of interest.

### The pandemic at Unimore

On 21 February 2020 the Coronavirus had just begun to spread outside China and the first outbreaks of the virus were detected in the North-East of Italy. Two days later, on February 23, due to the dynamics of the virus in the neighborhood, the Emilia-Romagna Region imposed a four days closure of the activities to all the universities in its territory, thus including Unimore. This has been the first restrictive measure involving educational institutions, which will anticipate all other restrictive measures, including the first large-scale red zone, the one that the following week was imposed to the territory of the Modena province (i.e. the Italian name for the NUTS-3 region level). Indeed, since the virus spread over, the next week lessons did not turn back to in presence and the closing measures were instead extended to all Italian Universities as early as March 4, according to restrictive measures that will last until the summer.

In the Italian university system, the yearly activity is divided into two semesters, with lessons taking place from late September to December for the first semester and from late February up to the end of May in the second one. Consequently, the closure of the in-presence activities at Unimore, coincides exactly with the beginning of the second semester of the academic year 2019/2020. As a result, the shift toward remote learning at Unimore, unlike in the case of the other universities, has completely covered the semester affected by the first stage of the pandemic.

After the first wave of the virus, most Italian universities opted for solutions allowing at least a partial resumption of in-presence activities for the following semester. Unimore, instead, adopted a very restrictive policy announcing already in May 2020 that the activities would have remained in remote for all the first semester of the following academic year (i.e. 2020/2021) and that it would have been possible to attend the lessons remotely in the second semester of the following academic year independently from the evolution of the pandemic. The lessons turned back in presence only at the end of the second semester of the academic year 2020/2021and only for the first-year students. The latter decision, taken in December 2020, was driven by the fact that a second wave of Covid-19 contagions was in place during that period and a third wave was largely expected for the successive months. In Italy, to be noted, the first wave of Covid-19 contagions took roughly place from February to May 2020, the second wave from October to December 2020, and the third wave from February to April 2021.

Following the timing of the main waves of coronavirus contagions, the pandemic period can be split in three different sub-periods in the Unimore context. The first one arrives up to September 2020 and corresponds to the first wave of contagions, the complete shift of the University activities to remote mode, and to the national restrictive measures. The second period, from October 2020 to March 2021, was characterized by the fact that Unimore was still closed and lockdown measures took a regional level dimension using a four colors classification. According to this new mechanism, the tightening of restrictive measures was based on a set of indicators at the regional level—mostly related to pressures of Covid-19 contagions on the healthcare system—which distinguished white, yellow, orange and red zones.. The third period, from April 2021 onwards, was instead characterized by a partial return to in-presence activities at Unimore thanks to a progressive loosening of social distancing measures and the massive vaccination campaign.

As for the scheduling of exams, whose grades are the outcome variable we are going to consider, in line with the other Italian universities, Unimore provides three regular sessions of exams: the winter session, from the beginning of January up to the end of February; the summer session, spanning from the half of May to the end of July; and the fall session, from the end of August to the end of September. According to the specific course, there are also a number of cases where exams are held in extra-ordinary sessions (April to May and October to December). The first exams in the Covid-19 period are thus the ones in April 2020, the last exams of the first sub-period ends with the exams of the fall 2020 regular session, the second sub-period starts with the extra-ordinary sessions of October and December 2020, includes the 2021 winter session end finishes with the exams of the extraordinary session in spring 2021, the last period covers the regular sessions of summer and fall 2022.

### The Unimore dataset

This study relies on Unimoredata, a database created with a specific Unimore project integrating all students’ individual information from administrative records and many large scale surveys (e.g. the Almalaurea post-degree surveys on early access to the labour market) since 2001.

Specifically, for the purpose of the presented analysis, we refer to a dataset merging together detailed information from the following administrative archives: (1) the register containing demographic characteristics of each student; (2) the archive reporting yearly information on each Unimore course attended by each student; and (3) the archive collecting all exams passed by each student attending Unimore. The latter dataset is particularly important for our analysis, as it contains full information about students’ passed exams, like the obtained mark, the date of notification, the subject, the teaching period, and teachers’ characteristics. According to the administrative data collection policies in Italian public Universities, failed exams are instead not recorded. Further investigation, however, have shown that during the pandemic the dynamics of passed exams had very a similar path to those of average exams marks which, as we will see below, have slightly increased. At same time, drop out rates increased by 2.1 percentage points, showing thus very similar patterns as those record elsewhere as in ^9^.

The analysis focuses on the grades of passed exams held in the period ranging from January 2018 to September 2021, thus our reference period starts from more than two years before the pandemic and then covers all the period characterized by the first three and major waves of Covid-19. We decide to restrict the sample of analysis considering only students aged 18–36 years old. Despite students being 37 years old or more represent a clear minority group (about 2% of the sample), we choose to exclude them from the analysis because their peculiar characteristics makes overall unclear their condition during the pandemic (e.g. they may be employed in remote working or in layoff/furlough period). Due to similar reasons, we also drop from the sample those students who still haven’t held any exam one year after the standard end of the course (about 5% of the sample). We also drop the exams for which we miss information about the teacher since they correspond to courses taught by teachers who are recruited on annual contracts and thus normally change from year to year (about 9.5% of the sample). In conclusion, our analysis relies on a sample of 371.000 exams held and passed by about 38,000 students. A detailed description of all variables used in the analyses and main descriptive statistics on the sample of students are presented in the Supplementary Material (Supplementary Table S1 and Table S2 respectively).

In the second part of the analysis, we build a difference-in-differences (DID henceforth) identification strategy exploiting also the information about the courses held with mix modality of teaching. However, as the provision of such kind of courses is not common to all departments, we exclude from the sample of analysis all observations referring to departments where these course are not supplied. With this last sample restriction the second part of the analysis relies on about 230 thousand exams. Also the main descriptive statistics on this reduced sample of students are presented in the Supplementary Material (Supplementary Table S3).

## Econometric strategy

The performance of students exams is analyzed by looking at the mark of each single exam as resulting from the administrative archives.

The benchmark model uses the following linear specification:

1 $$y_{j,i,t} = \alpha + \beta X_{i,t} + \delta m_{t} + \gamma Z_{j,t} + \theta C_{t} + \varepsilon_{j,i,t}$$

where ${y}_{j,i,t}$ is the mark obtained at the *j* exam of the student *i* at time *t* (if the student attends and passes the exam); ${X}_{i,t}$ and ${Z}_{j,t}$ are two vectors respectively of student level and exam level controls (some of them are time varying); ${m}_{t}$ is the month of the exam; ${C}_{t}$ is the dummy variable for the Covid-19 period, that is set alternatively as a single dummy or a set of dummies distinct by the 3 sub-periods outlined above, and ${\varepsilon }_{j,i,t}$ is the error term. The equation is estimated with linear OLS and errors are clustered at student level. The set of controls at student level ${X}_{i,t}$ includes: students’ demographic characteristics as gender, age, NUTS-3 region level region of birth and region of residence; the kind of upper secondary school attended before university (11 different categories); a dummy variable for being a sophomore or junior student and the number of exams already passed by the student at each exam date (i.e. proxies of students’ tenure and quality). The set of controls at the exam level ${Z}_{j,t}$ includes: the specific department of the degree program; a dummy for master degree courses (vs bachelor ones); the number of university credits (CFU) related to the exam; the exam month. To be clear, in the Italian system each exam correspond to an amount of credits varying from 3 to 12, and usually equal to 6 and 9; the greater is the number of credits the higher is the complexity and somewhat the difficulty of the exam. Formally, a CFU represents about 25 studying hours (in general assuming 7/8 h of lessons attendance and 17/18 h of ‘study at home’). A bachelor degree is generally reached after the completion of 180 CFU, while master degree courses count 120 CFU. Furthermore, we include in ${Z}_{j,t}$ also teachers individual fixed effects to account for this important source of heterogeneity, corresponding to 1160 dummy variables in the benchmark case.

In this benchmark model we thus focus on the coefficient $\theta$ representing the overall impact of the pandemic, similarly to what most of the literature outlined above does. As we discussed above, this approach would catch the effect on measured performance rather than to actual one. Thus, once set up this base model, we move to assess an identification strategy aimed to disentangle the effect of the changing teaching (and thus learning) methodologies first, and then the effects of the exposure to restrictive measures.

### Identifying the impact of (suddenly) changing teaching models

In this section we set up an econometric strategy to identify the impact of the shift from in-presence to remote learning brought about by social distancing measures. Thus, we are not going to evaluate the effectiveness of different teaching methodologies in normal times, we are instead analyzing the impact of a forced sudden shift that has also often caught unprepared teachers and technical staff.

As we have anticipated in previous sessions, possible negative effects on students’ actual performance could be overshadowed by opposing changes in measured performance related to changing examination modes. To avoid student misconduct, and in compliance with the general directives of the Italian Ministry of Education, Unimore adopted a set of arrangements to the remote examination modes that included student room control systems, software to control the activities of the personal computers used for examination tests (Safe Exam Browser), and limits to the ratio of examining students to teachers assigned to video surveillance. Such arrangements have reduced possible misbehavior however surely not eliminated it. At same time, the same shifts of exams modes with this related arrangements might have impacted on students’ performance during the exam. An analysis of the impacts on actual student performance, therefore, cannot disregard all this performance measurement problems. To this end, we will set up a DID identification strategy relying on the fact that while the shift in exams mode, with the related performance measuring biases, has equally concerned all courses, the change in teaching modalities has not been equal for all. Indeed, many Departments at Unimore, before the pandemic, already included in their supply degree programs with an hybrid online and in presence learning. In these programs all courses have only a share of teaching using traditional face to face methodology, and this share corresponds on average to the half of the course teaching activities with very little variation among courses. At same time, this courses have all same in-presence evaluation modes independently from the teaching modality. Thus, the shut off of in-presence activities had different consequences in terms of intensity in changing teaching modes among hybrid and standard courses but same consequences in terms of changes in evaluation modes and then also in performance measuring standards. In particular, we can argue without loss of generality that the impact in terms of changing teaching methodologies was double in the case of standard in-presence programs respect to hybrid ones.

We exploit this option in a DID approach by adding to the base specification in Eq. (1) the course modality variable and its interaction term with the Covid-19 variable:

2 $$y_{j,i,t} = \alpha + \beta X_{i,t} + \delta m_{t} + \gamma Z_{j} + \theta C_{t} + \mu D_{j} + \pi C_{t} \cdot D_{j} + \varepsilon_{j,i,t}$$

where the variable ${D}_{j}$ is a dummy representing the course modality in normal times: in-presence (${D}_{j}=1$) or hybrid one. A negative sign of the interaction coefficient $\pi$ would evidence a relatively worst performance for exams in traditional programs respect to those in hybrid ones and thus, according to the DID strategy interpretation of causal inference, supporting for a negative impact of the shift to distance teaching. Moreover, since the teaching modality shift is double for courses in standard programs respect to those in hybrid programs, in terms of magnitude we can state that the impact estimated is a lower bound estimation that should correspond to half of the actual impact.

In the sample of analysis, these hybrid courses represent around 19% of the students and the online teaching usually represent half of the classes for each exam. As students and exams could have different features in the two kind of programs, we correct for possible composition biases by using an Inverse Probability Weighting (IPW) strategy with the hybrid mode variable *D* as treatment variable. The IPW estimate relies on the following set of covariates: students’ demographic characteristics (i.e. gender, age, and NUTS-3 level region of birth and residence); the kind of upper secondary school; the year of enrollment; the specific department; and a dummy for master degree courses (vs bachelor ones). Finally, to properly isolate the effect of changing in teaching modes, we restrict the sample to the exams corresponding to classes taught in the immediately preceding teaching period (i.e. about 161 out of 223 thousand of exams). In fact, exams can be attended either in the months immediately following the end of classes but also in next semesters, several months after. We limit our analysis to the former case of ‘on-schedule’ exams. With this sample restriction we narrow the analysis on exams prepared by students attending courses taught according the modalities corresponding to the same specific period (before and after Covid-19 and also, in case, to the specific sub-periods). Moreover, by doing so, we can focus on exams whose preparation is more strictly related to the classes attendance rather than to the use of supplementary materials, such as handbooks or slides.

### Identifying the impact of the exposure to restrictions

To identify the effect of exposure to restrictive and lockdown measures, we exploit the consequences of the very prudential policy implemented by Unimore about the recovery of normal activity described above.

As elsewhere, the closure of universities led to the return to their origin places of a large part of students being not resident in the neighborhood of the University. Suggestive is the case of Milan, where the news of the regional lockdown for the following day, circulated in advance because of a communication mistake, caused an exodus of students from North to South Italy so massive as to strongly impact on the spread of the virus in the southern regions of the Country while it was still concentrated only in the Northern regions.

During summer 2020, while Covid-19 related restrictions had been loosened by the national government, the universities were allowed to decide autonomously whether to re-start in presence activities for the next year. The decision in most campus or university cities contexts to reactivate in-presence activities, with the need to bear the cost of new infrastructure needed to respect legal prescriptions for social distancing, have also been driven by the economic interests of the neighborhood, for which the closure of the university leads to significant losses, like in the case of the owners of rental properties, commercial activities, and so on.

This was not actually the case for Unimore. In this area, the university has indeed a significant impact on its territory, but the economic vocation is another, ranging from automotive (Ferrari, Maserati, etc.) to food processing (e.g. Parmigiano Reggiano, Modena’s Balsamico), via robotics and ceramics. Moreover, the Modena city hospital, which was among those most put under pressure since the first waves of the pandemic, is part of the same university and has significant political weight even in the managerial offices (the same chancellor was a professor of the department of Medicine). As a consequence, the subjective experiences of professors and other civil servants grounded in departments operating within the Modena hospital understandably had a weight on their attitudes on the level of precautions to take.

As a result, Unimore adopted different decision respect to most universities, as the neighboring University of Bologna, which guaranteed a reopening of activities also through ad hoc investments for mixed teaching and the intervention of public institutions providing housing supports for students. Just before the end of the second semester of 2020, Unimore finally announced that the activities of the first semester of the following academic year—starting in September 2020—would have kept the distance mode. This exacerbates the emptying of the cities of Modena and Reggio Emilia, as evidenced by the attention given by the local press. Indeed, since then, also for the contribution of the very high living costs characterizing the cities of Modena and Reggio Emilia, most students returned to their homes and freshmen did not come in Modena and Reggio Emilia to find a new accommodation. This depletion is also confirmed by the fact that at the end of the second semester of the 2021/2022 academic year, when in-person attendance was reopened for a number of courses, despite the announcement made well in advance, only a minority share of students actually returned physically to the classroom while the rest continued to attend remotely. This decision did not turn out to be so wrong if one considers that the arrival of the second and third waves of the virus also induced the other universities to close down again.

**Figure 1:**
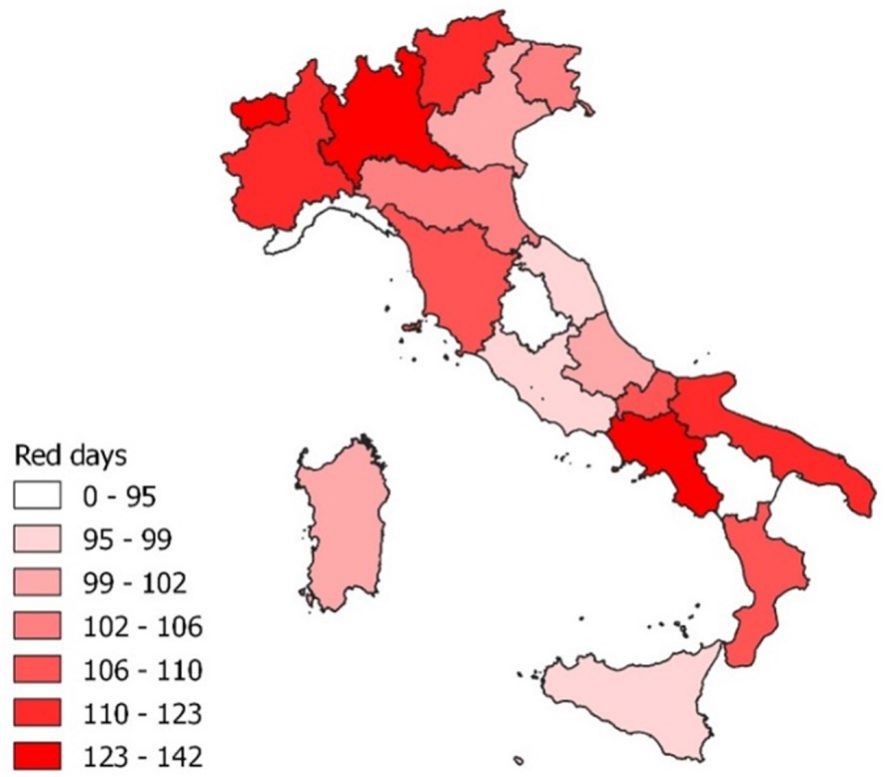
Cumulated number of days in red classified regional conditions.

At the same time, restrictive measures took a regional articulation from October 2020, following the four-color classification mentioned above. This induced a strong heterogeneity in students exposure to restrictions. The restrictions adopted in the case of red classification are similar to the lockdown implemented nationwide from March to May 2020, thus an overall home confinement. Accordingly, the time-varying restrictions in place at the residence of each student are a reliable proxy of the restrictions to which she has been subjected having a relevant time and space varying dimension. Figure 1 gives evidence of the regional heterogeneity of the cumulated restrictions from the beginning of the pandemic to September 2021, but the time-varying dimension of restrictions is relevant as well. To be noted, for the sake of the analysis, the national level lockdown imposed during the first wave of the virus, which lasted 70 days, is considered as a red zone and included on each regions’ records.

We exploit this peculiarity to analyze two different aspects of the exposure to the restrictive measures. First, we consider the impact of cumulated exposition to restrictive measures since the start of pandemic. Second, we consider the effect of exposure to restrictive measures during the exam preparation period. To do all this, we add to the benchmark model in Eq. (1) one variable in two different cases. For each date of exam, in the first case we compute the cumulated number of days that the region of residence has passed under red zone restrictions while in the second one we compute the share of days in red zone over the 14 days before the exam. As we count among the days spent in a red zone also those related to the national level lockdown, when these variables still have a time-varying dimension and then allow for some heterogeneity, we can use all the data period from May 2020 onwards.

## Results

In detailing our findings, we start by providing an overall picture of students’ performance after and before the Covid-19 pandemic in Table 1. In the first column we report estimates of the model specification presented in Eq. (1) and, in particular, the coefficient of the Covid-19 dummy variable being 1 for the whole period ranging from April 2020 to September 2021. The coefficient is positive and significant at 1% level.

**Table 1:** Impact of the Covid-19 period on exams’ mark.

	Full period	Sub-periods	First year
Covid	0.186***		0.224***
Covid I		0.233***	
Covid II		0.221***	
Covid III		0.078***	
Observations	370,955	370,955	319,058

Standard errors are clustered at student level. *p < 0.1; **p < 0.05; ***p < 0.01. While only coefficients of the variables of interest are presented here, all estimates are based on a model specification including covariates listed in “Econometric strategy” section.

In terms of magnitude, considering that exam marks at the Italian universities are expressed over 30 points with 18 being the minimum of passed exams and the standard deviation in the sample is 3.7 points, the value of 0.186 reported in the first column of Table 1 is not negligible although low. When we look at the three sub-periods of pandemic discussed in “The pandemic at Unimore” section separately, the coefficient is still positive and significant for each sub-period (second column of Table 1). The positive effect is concentrated in the first two periods of the pandemic, where the coefficient is a bit greater than 0.2. In the third period (i.e. April-September 2021), the coefficient becomes much lower but it remains still significant. The lower magnitude of the coefficient in the last period is consistent with the partial reopening of in-presence activities, which could blur the pandemic influence on the students’ performances. To account for this possible confounding factor, in the third column of Table 1, we report the estimate of the overall impact limited to the first year of the pandemic only, thus limiting the reference period to April 2021 rather than September 2021. In this case the coefficient of the Covid-19 dummy variable has a value close to those reported in the first two sub-periods of pandemic.

In conclusion, the evidence provided in Table 1 would suggest that in relation to the sample of passed exams, students’ performance has slightly benefited from the pandemic, consistently with other studies of the literature surveyed above which use the same Covid-19 period dummy variable approach or else rely on some descriptive evidence. Our explanatory hypothesis, that we attempt to confirm in what follows, is that this unexpected outcome is mainly driven by a misalignment between the reported performance and the actual one. Indeed, because of the shut off of all in-presence activities, not only classes but also the exam evaluation shift to remote, becoming more slack. (To be clear, we are not able to assess whether these changes in the evaluation standards are due to a change in the kind of exams made—which also shifted from in-presence to remote—or to the adoption of magnanimous criteria by teachers.) This hypothesis could also fit with the partial different behavior of the last period, when time elapsed and experience cumulated could have impact on the effectiveness of assessment modes.

In what follows, we go beyond the analysis of the overall effect on reported performance to explore the two main different channels through which the pandemic may have negatively impacted actual performances: the sudden shift to remote teaching and the home confinement.

### The impact of (suddenly) changing teaching models

Table 2 shows the estimation results of the model specification presented in Eq. (2) and corresponding to the identification strategy outlined in “Identifying the impact of (suddenly) changing teaching models” section. This strategy is aimed at disentangling the effect of the sudden shift to remote teaching on students’ performances. To do that, as anticipated in “Identifying the impact of (suddenly) changing teaching models” section, we first restrict the sample to the departments having both in-presence and hybrid courses (see “The Unimore dataset” section), then consider only the exams corresponding to classes taught in the immediately preceding teaching period, and then estimate the IPW weights using the course modality as treatment variable.

**Table 2:** Impact of the Covid-19 on exams’ mark, interaction with courses’ teaching modality.

	(1)	(2)	(3)	(4)	(5)	(6)
	Base model full period	DID model full period	Base model first year	DID model first year	Base model sub-periods	DID model sub-periods
Covid	0.277***	0.769**	0.368***	1.222***		
Covid I					0.318***	1.078***
Covid II					0.392***	1.207**
Covid III					0.056	− 0.112
In-person	− 0.347***	− 0.125	− 0.491***	− 0.189	− 0.348**	− 0.120
In-person*Covid		− 0.608**		− 1.043***		
In-person*Covid I						− 0.917***
In-person*Covid II						− 0.990***
In-person*Covid III						0.166
Observations	161,058	161,058	139,442	139,442	161,058	161,058

Standard errors are clustered at student level. *p < 0.1; **p < 0.05; ***p < 0.01. While only coefficients of the variables of interest are presented here, all estimates are based on a model specification including covariates listed in “Econometric strategy” section.

Column 1, 3 and 5 of Table 2 presents the same base model shown in the previous section restricted to departments providing at least one hybrid course and with the addition of a control variable for the course modality (1 if in-person and 0 otherwise) and using the IPW correction (see Supplementary Table S4 for the first stage estimations). To be noted, Supplementary Table S5, which is the equivalent of Table 1 in the subsample used in this IPW case, highlights that the pandemic-related coefficient does not change much with respect to the one presented in Table 1. This evidence confirms that the sample restrictions here adopted, as well as the bias on the coefficient of variables not related to the IPW treatment variable due the application of the IPW correction, does not affect significantly our results. In column 2, 4 and 6 of the same table we use the DID specification presented in “Identifying the impact of (suddenly) changing teaching models” section.

In the baseline case, exam marks of students attending in-presence courses are lower if compared to those reported by students attending hybrid courses. When we consider the DID model which adds the interaction term, however, the effect of attending in-presence courses is not significant anymore while the coefficient of the interaction term is negative and strongly significant. Columns 3 and 4 of Table 2 present the same analysis shown in Columns 1 and 2 limiting the reference period to the first year of pandemic (i.e. up to May 2021), thus focusing on the period during which all classes were attended remotely. Clearly, in this case, the magnitude of the interaction term is much larger than before (1.0 vs 0.6 points), as well as the one of the Covid-19 dummy (1.2 vs 0.3 points).

Summing up, the hypothesis according to which the sudden shift to remote teaching had negatively affected students’ performance finds evidence in our results. As hybrid courses generally have half of lessons in presence, we can estimate the total impact of the change in teaching modality by doubling the coefficient of the interaction term, and thus obtaining a value of about 2 points out of 30. To better understand the extent of the estimated effect related to the pandemic, it should be considered that this value represents more than half of the standard deviation of exam marks and 6.6% of the overall marks range. Our estimated value of the losses related to the shift to remote teaching is close to the upper threshold of the results provided by ^9^ although obtained with different econometric set-up, unit level analysis, performance outcome and in a case study of another country (US vs Italy).

At same time we also confirm the hypothesis that changes in assessment modes are prominent drivers of the increase in student reported outcomes evidenced in the literature. This effect has offset the negative impact of the pandemic period misaligning the effective performance of students from the measured one. Indeed, when we shift from the base to the DID specifications the coefficient of the Covid-19 variable increases substantially and to an extent close to the absolute value of the coefficients of the interactions included.

### The impact of the exposure to restrictions

We move now to the analysis of the impact of the exposure to mobility restrictions on students’ performances described in “Identifying the impact of the exposure to restrictions” section. To do this purpose, we slightly restricts the sample of the benchmark case (see Table 1) as we exclude the exams held by students who are not resident in Italy (they represent less than 2% of the full sample of exams). The second column of Table 3 adds to the base model—whose results are reported in column 1—the overall number of days each student spent under red zone restrictions, while the third column adds the variable reporting the share of days spent under red zone restrictions over the 14 days before the exam. As explained above, in the former case we focus on the cumulated impact of restrictions, while in the latter we assess the impact of being confined at home in the days just before the exam’s session, corresponding to the period of exams’ preparation.

**Table 3:** Impact of mobility restrictions on exams’ mark.

	Full period	Full period	Full period
Covid	0.186***	0.432***	0.169***
1 day more in red zone		− 0.003***	
% red zone last 2 weeks			0.176***
Observations	370,955	368,501	368,501

Standard errors are clustered at student level. *p < 0.1; **p < 0.05; ***p < 0.01. While only coefficients of the variables of interest are presented here, all estimates are based on a model specification including covariates listed in “Econometric strategy” section.

Table 3 highlights that the number of days spent under red zone restrictions decreases the exam marks. One day more spent under lockdown restrictions corresponds to a reduction of 0.003 points. Considering that at the end of the reference period the average value of this variable is 105 days, we can estimate the average effect on students’ exam marks at the end of the pandemic to be about one third of point. At same time, as also in the previous section, when we take into account this negatively impacting channel, the estimated coefficient of the Covid-19 dummy increases.

While the results shows a negative long run effect of home confinement, that can be ascribed to mental stress issues, when we look at the effect in the short run, things substantially change. Our results show that a greater number of days spent under red zone restrictions during the two weeks preceding the exam (i.e. probably those on which the preparation to the exam is mainly concentrated) engenders an increase of students’ exam marks. In this case, the Covid-19 dummy coefficient does not report any relevant variation, confirming the change of examination modality to represent the main explanation of the positive impact on measured performance.

In conclusion, the results of our analysis suggest a composite effect of mobility restrictions. On the one hand, consistently with the results provided by ^6^, they might have increased the amount of time allocated to study for exams thus improving performances in the short run. On the one other hand, however, in the long run the protracted exposure to the restrictions clearly reduced the students’ outcomes.

## Robustness checks

In this session we present two different robustness checks, one for each of the two channels we considered in main analysis: the change in teaching modalities and the exposure to mobility restrictions.

As for the change in teaching methods, we perform a placebo test analysis. Instead of restricting the sample to on-schedule exams only, we consider the other exams: those made during the pandemic but related to courses attended in the pre-Covid-19 semesters. This test should therefore be considered as valid if two conditions hold. The first one is that the coefficients of the baseline model of the Covid variables are still positive and significant. This would confirm the increase in exams grade is due to the change in exams modality and not to change in teaching modes. The second condition requires that in the DID specification the coefficients of the interaction term between the Covid-19 dummy and the in-presence course one are found to be insignificant or to have very small magnitude. Results of the placebo test, presented in Table 4, confirm the robustness of our results. In fact, while in the baseline model the coefficients of the covid variables confirm the baseline specification results, the coefficients of the interaction terms in the DID specification are always insignificant and their magnitude is strongly reduced if not even with opposite sign if compared to those reported in Table 2.

**Table 4:** Interaction with courses’ teaching modality, different semester.

	(1)	(2)	(3)	(4)	(5)	(6)
	Base model full period	DID model full period	Base model first year	DID model first year	Base model sub-periods	DID model sub-periods
Covid	0.131*	0.086	0.177**	0.438		
Covid I					0.231**	0.508
Covid II					0.129	0.743
Covid III					0.027	− 0.571
In-person	− 0.325	− 0.340	− 0.329	− 0.264	− 0.318	− 0.304
In-person*Covid		0.054		− 0.307		
In-person*Covid I						− 0.342
In-person*Covid II						− 0.634
In-person*Covid III						0.698
Observations	61,043	61,043	50,875	50,875	61,043	61,043

Standard errors are clustered at student level. *p < 0.1; **p < 0.05; ***p < 0.01. While only coefficients of the variables of interest are presented here, all estimates are based on a model specification including covariates listed in “Econometric strategy” section.

As for the effect of restrictions on students’ performances, one possible weakness of our strategy is the fact that some students may not have returned back to their households and thus the restrictions in place in the region of origin may not correspond to the actual restrictions to which these students where subject to. This would affect our estimates but only partially since in the first stage of pandemic, the variables of interest have only a time variation, not spatial, because restrictions had national dimension. As to the following period, the option of not coming back home does not apply to freshmen students since the decision to keep university activities in remote mode for all the first semester, and to allow in any case to attend classes in remote for all the rest of the year, was communicated well before the opening of course registration. Besides, for the same reasons the case of not coming back home even after the first Covid-19 wave is less likely to have occurred for non-freshmen students because of the rent costs that could be saved. To be noted, house rent costs in Modena and Reggio Emilia are indeed particularly high if compared to other university cities as recorded by the yearly official statistics on living costs performed by the Italian Institute of Statistics, which places the two cities among the highest in Italy for living costs. Finally, it is likely that the climate of fear and concern that had spread in the early stages of the pandemic pushed most of people returning to their household of origin just before the end of the first national lockdown in May 2020 independently from the high economic incentives.

Anyway, to account for this possible source of bias we perform a sensitivity analysis by restricting the sample to students resident out of the Modena and Reggio Emilia provinces. We consider only students who faced the same decisions about where to spend the periods of suspension of university in-person activities, thus the bias would affect randomly all kind of students. Table 5 highlights that the coefficients of variables regarding the effect of restrictive measures do not change substantially with respect to those reported in Table 3, overall confirming the robustness of our main results.

**Table 5:** Impact of mobility restrictions on exams’ mark.

	Full period	Full period	Full period
Covid	0.170***	0.425***	0.143***
1 day more in red zone		− 0.003***	
% red zone last 2 weeks			0.267***
Observations	149,371	146,917	146,917

Students being resident out of Modena and Reggio Emilia provinces only.

Standard errors are clustered at student level. *p < 0.1; **p < 0.05; ***p < 0.01. While only coefficients of the variables of interest are presented here, all estimates are based on a model specification including covariates listed in “Econometric strategy” section.

In the Supplementary Material we also report a heterogeneity analysis of our main results (i.e. those in Table 1 and Table 2) to assess whether they present any relevant change when distinguishing departments by ERC sector or teachers by age group (aged 59 or younger vs aged 60 or older). Specifically, Supplementary Table S6 and Table S8 show the heterogeneity of the Covid-19 impact on students’ exam marks by ERC sectors, while Supplementary Table S7 and Table S9 do the same by teachers’ age group.

Supplementary Table S6 points out that coefficients in the first column and the last column always have the same statistically significance and direction. As for the magnitude, departments in the Life Sciences sector (e.g. Medicine and Nursing) seem the most affected by Covid-19, while coefficients of Social Sciences and Humanities and STEM sectors are very similar each other. These results are overall confirmed in the analysis by pandemic period with the exception of Social Sciences and Humanities departments, where the coefficient for the Covid III period is positive but insignificant (in line with results in Table 2 though). Moreover, while the in-presence students appear to have different performances by ERC sector, Supplementary Table S8 highlights that the Covid-19 effect related to the change of teaching modality is negative and significant in all departments except for those in Life Sciences. As for the heterogeneous effects by teachers’ age group, Supplementary Table S7 shows that coefficients are very similar, then suggesting that older teachers have not behaved differently from others. Nonetheless, the DID analysis in Supplementary Table S9 points out a heterogeneous causal effect of Covid-19, which is significant only for the subgroup of older teachers when considering the full period (column 2). This evidence seems to suggest that teachers’ reaction to pandemic-related changes was similar during the first year of pandemic, but the effect has lasted longer among older teachers. All in all, they were more vulnerable to the COVID disease and probably have had a harder time to adapt to the online modality.

Finally, Supplementary Table S10 presents a robustness check on the overall effect of the time spent in a regional red zone during the two weeks preceding the exam (see Table 3). First, we provide an estimation where Covid-19 period dummies are included. Second, we provide an estimation focusing on the first year of pandemic only, to assess whether the effect estimated for the full period is stable or not over time. Supplementary Table S10 clearly shows that the effect of the variable of interest here is slightly lower than the one presented in Table 3 (differences are not significant at 10 percent level though), but still strongly positive and significant. This evidence confirms the effect of being forced at home during the two weeks before the exam is actually quite stable over the analyzed period.

## Conclusions

In this study, we have focused on the effect of the pandemic on the performance of university students. By exploiting the opportunities provided by an administrative dataset containing very detailed information on the University of Modena and Reggio Emilia (Unimore), one of the forerunners of the restrictions imposed worldwide to universities during Covid-19 early stages, we have tried to solve some inconsistencies in the literature and to unbundle the two main channels through which the pandemic changed university students’ pathways: the shift to remote lessons and the exposure to lockdown measures.

On the one hand, the results of the DID estimations based on the distinction between full in-presence programs and hybrid ones suggests a mismatch between actual performance and measured performance related to the change in assessment methods and/or parameters. In the standard design that uses the Covid-19 period as treatment, the evidence is that of an overall albeit slight improvement in average marks: in the context of a grading system with marks expressed in thirtieths, with 18 as the minimum grade of passed exams and a variance of 3.6, during the pandemic the score of passed the exams increased by one sixth of point, a result substantially in line with that of the literature which also shows slightly positive overall effects in a number of different outcomes of students’ performance. Besides, to a more detailed insight, the pandemic still appears to have had negative effects on student performance. The evidence gathered allows us to estimate the impact of the sudden change in lecture modes in nearly two thirtieths. This result is in line with the literature focusing on specific aspects of the Covid-19 impact and also coherent with the studies on students’ subjective evaluations. Despite this channel seems to have been more relevant, also the psychological effects due to exposure to lockdown measures result as significant: at the end of the period considered, the cumulative impact of exposure to home confinement amounting to about one third of point. At the same time, being confined at home in the two weeks prior to the examination date appears to have had a positive impact: being forced to stay at home during all the two weeks before the exams increase the average grade by nearly one sixth of point. Nonetheless, the driver of the overall positive effect on students’ grades seems to be the change in evaluation standard, that result in having increased student grades by a value in the range of 2–2.5 thirtieths.

As a result, if we look at the effect on student’s actual performance, and thus on their process of human capital accumulation, we can support the evidence of an appreciable negative impact that has been, however, offset on the surface by an average more slack assessment systems. This gives rise to two different kind of problems. The first concerns the most well-known and direct aspect: the loss in terms of human capital accumulation, a significant loss that might have long-term effects. There is however also a further aspect. This generation of students will turn out to be less prepared compared to the others, regardless their similar average marks. This, over time, could produce a stigma effect by fostering a widespread perception that those who studied in the pandemic years are less capable if compared with other ones with same degree or marks. While this may be true for some, in particular for those who have benefited most from the different assessment modes, it is not true for all. Anyway, the signaling role of their degree on job applicants would be weakened. This could result in a process of statistical discrimination: an efficient practice for those who implement it, the employers, but as unfair for an already hard-hit generation of students.

### Supplementary Information

Supplementary Tables.

## Data Availability

The datasets generated and analyzed during the current study uses the information coming from the administrative archives of the University of Modena and Reggio Emilia. They are not publicly available due restrictions related to data ownership but they are available together with all do files from the corresponding author on reasonable request by remote connection to a dedicated server. The research did not rely on any kind of experiments on humans and/or the use of human tissue samples. The whole research was performed in accordance with relevant guidelines/regulations, in particular with all requirements imposed by the Italian Data Protection Authority (GDPR) in its November 27, 2008 Requirements (Gazzetta Ufficiale No. 300, December 24, 2008) and subsequent and possible adjustments and amendments. In compliance with Regulation (EU) 2016/679 of the European Parliament and of the Council of April 27, 2016, Legislative Decrees August 10, 2018 No. 101 and May 18, 2018 No. 51 of the Italian Government, the study did not required ethics approval and/or individual consent of the involved persons (the students of Unimore), who, in any case, at the time of matriculation at Unimore were informed about the processing of personal data also for purposes that respond to and are aimed at implementing the exercise of institutional powers vested in the university, including research.
