# Supplementary material for: Unraveling the controversial effect of Covid-19 on college students’ performance
Source: Sci Rep. 2023 Sep 23;13:15912. doi: 10.1038/s41598-023-42814-7 (PMC10517930; doi:10.1038/s41598-023-42814-7)
Supplement: Supplementary file 1 — Supplementary Tables. [file 41598_2023_42814_MOESM1_ESM.docx]

# Supplementary Material

### Table S1. Variables description (Panel A)

| Variable | Description |  |
| --- | --- | --- |
|  |  |  |
| *Dependent variable* | |  |
| Exam mark | Continuous variable representing the mark of each exam. It ranges from 18 to 30. |  |
| *Variables of interest* | |  |
| Covid | Binary variable taking the value of 1 for all exams passed since March 2020 onwards, and 0 otherwise. |  |
| Pre-pandemic period | Binary variables representing the different phases of pandemic from its advent to September 2021. Specifically, Covid I represents the first wave of Covid-19 pandemic (i.e. from March 2020 to September 2020), Covid II refers to the second and third waves of pandemic (i.e. October 2020 to April 2021), and Covid III represents the post-vaccination period (i.e. from May 2021 to September 2021). The reference category is 'Pre-pandemic period' (i.e. from January 2018 to February 2020). |  |
| Covid I |  |  |
| Covid II |  |  |
| Covid III |  |  |
| In-person | Binary variable taking the value of 1 for students enrolled in an in-person degree course, and 0 otherwise (i.e. hybrid degree course). |  |
| On-schedule exam | Binary variable taking the value of 1 for those exams passed immediately after the teaching semester to which the course refers, and 0 otherwise. |  |
| 1 day more in red zone | Continuous variable counting the number of days passed by each student in pandemic-related red zone at the exam date. |  |
| % red zone last 2 weeks | Continuous variable reporting the share of days passed by each student in pandemic-related red zone during the two weeks immediately before the exam date. |  |
| *Independent variables* | |  |
| Female | Binary variable taking the value of 1 for women and 0 for men. |  |
| Age | Continuous variable representing the student's age. |  |
| Age squared | Continuous variable representing the student's age squared. |  |
| Born in MO or RE | Binary variables reporting the territory of birth of students. 'Born in MO and RE' means being born in the Modena or Reggio Emilia NUTS-3 regions. 'Born in ER region' means being born in the Emilia-Romagna NUTS-3 regions different from Modena or Reggio Emilia. 'Born in Italy' means being born in a NUTS-2 region different from Emilia-Romagna. Finally, students who were born abroad are divided according to whether they hold Italian citizenship. The reference category is 'Born in MO and RE'. |  |
| Born in ER region |  |  |
| Born in Italy |  |  |
| Born abroad and Italian |  |  |
| Born abroad and non-Italian |  |  |
| Resident in MO or RE | Binary variables reporting the territory of residence of students. 'Resident in MO and RE' means being resident in the Modena or Reggio Emilia NUTS-3 regions. 'Resident in ER region' means being resident in the Emilia-Romagna NUTS-3 regions different from Modena or Reggio Emilia. 'Resident out of ER region' means being resident in an Italian NUTS-2 region different from Emilia-Romagna or abroad. The reference category is 'Resident in MO and RE'. |  |
| Resident in ER region |  |  |
| Resident out of ER region |  |  |

### Table S1. Variables description (Panel B)

| Variable | Description |  |
| --- | --- | --- |
| *Independent variables* | |  |
| Classical Lyceum | Binary variables reporting the type of high school in which students are graduated. The reference category is 'Classical Lyceum'. |  |
| Scientific Lyceum |  |  |
| Linguistic Lyceum |  |  |
| Socio-educational Lyceum |  |  |
| Other Lyceum |  |  |
| Commercial school |  |  |
| Industrial school |  |  |
| Other technical school |  |  |
| Professional school |  |  |
| Other school |  |  |
| Foreign diploma |  |  |
| Sophomore or Junior year | Binary variable taking the value of 1 for a sophomore (second year) or junior (third or more year) student, and 0 otherwise. |  |
| Physiotherapy and Dental hygiene | Binary variables reporting the specific UNIMORE department in which students are enrolled. |  |
| Education and Human Sciences |  |  |
| Communication and Economics |  |  |
| Economics |  |  |
| Law |  |  |
| Engineering |  |  |
| Medicine and Nursing |  |  |
| Chemical and Geological Sciences |  |  |
| Physical and Mathematical Sciences |  |  |
| Life Sciences |  |  |
| Engineering Sciences and Methods |  |  |
| Language and Cultural Studies |  |  |
| Master student | Binary variable taking the value of 1 for students enrolled in a master degree course, and 0 otherwise. |  |
| Number of exams already passed | Continuous variable counting the number of students' exams passed up to the last exam date. |  |
| 6 CFU or lower | Binary variables reporting the number of university credits related to the exam subject. The greater is the number of credits the higher is the complexity of and the number of hours taught by the exam. |  |
| 7-9 CFU |  |  |
| 10 CFU or more |  |  |
| January | Binary variables reporting the month in which the exam is passed. Usually, the typical months for exams in Italy are January, February, June, and July. We group the other periods on the basis of the season of the exam sessions. The period between March and May is the spring session, the period between October and December is the fall session. August and September are the months committed to the repair exams. |  |
| February |  |  |
| March-May |  |  |
| June |  |  |
| July |  |  |
| Aug-Sep |  |  |
| Oct-Dec |  |  |
| Life Sciences (LS) | Binary variables reporting the ERC sector in which students are enrolled. Fields are distinguished among Life Sciences (i.e. Physiotherapy and Dental hygiene, Life Sciences), Social Sciences and Humanities (i.e. Education and Human Sciences, Communication and Economics, Economics, Law, Language and Cultural Studes), and PE (i.e. Engineering, Medicine and Nursing, Chemical and Geological Sciences, Physical and Mathematical Sciences, Engineering Sciences and Methods). |  |
| Social Sciences and Humanities (SH) |  |  |
| Mathematics, physical sciences, information and communication, engineering, universe and earth sciences (PE) |  |  |
| Teacher aged 59 or younger | Binary variables reporting the professor's age. 'Teacher aged 59 or younger' means that the professor is 59 years old or less. 'Teacher aged 60 or older' means that the professor is 60 years old or more. |  |
| Teacher aged 60 or older |  |  |

### Table S2. Descriptive statistics of the main sample

| Variable | Obs. | Mean | Std. dev. | Min | Max |
| --- | --- | --- | --- | --- | --- |
| Exam mark | 370,955 | 26.1 | 3.5 | 18 | 30 |
| Pre-pandemic period | 370,955 | 0.568 | 0.495 | 0 | 1 |
| Covid I | 370,955 | 0.157 | 0.364 | 0 | 1 |
| Covid II | 370,955 | 0.136 | 0.342 | 0 | 1 |
| Covid III | 370,955 | 0.140 | 0.347 | 0 | 1 |
| Female | 370,955 | 0.564 | 0.496 | 0 | 1 |
| Age | 370,955 | 22.6 | 2.5 | 18 | 36 |
| Age squared | 370,955 | 515.2 | 125.8 | 324 | 1296 |
| Born in MO or RE | 370,955 | 0.514 | 0.500 | 0 | 1 |
| Born in ER region | 370,955 | 0.097 | 0.296 | 0 | 1 |
| Born in Italy | 370,955 | 0.333 | 0.471 | 0 | 1 |
| Born abroad and Italian | 370,955 | 0.019 | 0.138 | 0 | 1 |
| Born abroad and non-Italian | 370,955 | 0.037 | 0.188 | 0 | 1 |
| Resident in MO or RE | 370,955 | 0.597 | 0.490 | 0 | 1 |
| Resident in ER region | 370,955 | 0.104 | 0.305 | 0 | 1 |
| Resident out of ER region | 370,955 | 0.299 | 0.458 | 0 | 1 |
| Classical Lyceum | 370,955 | 0.078 | 0.268 | 0 | 1 |
| Scientific Lyceum | 370,955 | 0.378 | 0.485 | 0 | 1 |
| Linguistic Lyceum | 370,955 | 0.113 | 0.317 | 0 | 1 |
| Socio-educational Lyceum | 370,955 | 0.056 | 0.230 | 0 | 1 |
| Other Lyceum | 370,955 | 0.052 | 0.222 | 0 | 1 |
| Commercial school | 370,955 | 0.108 | 0.310 | 0 | 1 |
| Industrial school | 370,955 | 0.088 | 0.284 | 0 | 1 |
| Other technical school | 370,955 | 0.056 | 0.229 | 0 | 1 |
| Professional school | 370,955 | 0.036 | 0.186 | 0 | 1 |
| Other school | 370,955 | 0.018 | 0.135 | 0 | 1 |
| Foreign diploma | 370,955 | 0.017 | 0.128 | 0 | 1 |
| Sophomore or Junior year | 370,955 | 0.631 | 0.482 | 0 | 1 |
| Physiotherapy and Dental hygiene | 370,955 | 0.043 | 0.204 | 0 | 1 |
| Education and Human Sciences | 370,955 | 0.118 | 0.322 | 0 | 1 |
| Communication and Economics | 370,955 | 0.120 | 0.325 | 0 | 1 |
| Economics | 370,955 | 0.128 | 0.334 | 0 | 1 |
| Law | 370,955 | 0.077 | 0.267 | 0 | 1 |
| Engineering | 370,955 | 0.138 | 0.345 | 0 | 1 |
| Medicine and Nursing | 370,955 | 0.115 | 0.318 | 0 | 1 |
| Chemical and Geological Sciences | 370,955 | 0.029 | 0.168 | 0 | 1 |
| Physical and Mathematical Sciences | 370,955 | 0.029 | 0.167 | 0 | 1 |
| Life Sciences | 370,955 | 0.073 | 0.260 | 0 | 1 |
| Engineering Sciences and Methods | 370,955 | 0.068 | 0.253 | 0 | 1 |
| Language and Cultural Studies | 370,955 | 0.062 | 0.241 | 0 | 1 |
| Master student | 370,955 | 0.364 | 0.481 | 0 | 1 |
| Number of exams already passed | 370,955 | 13.56 | 10.59 | 0 | 100 |
| 6 CFU or lower | 370,955 | 0.461 | 0.498 | 0 | 1 |
| 7-9 CFU | 370,955 | 0.391 | 0.488 | 0 | 1 |
| 10 CFU or more | 370,955 | 0.148 | 0.355 | 0 | 1 |
| January | 370,955 | 0.190 | 0.392 | 0 | 1 |
| February | 370,955 | 0.170 | 0.376 | 0 | 1 |
| March-May | 370,955 | 0.086 | 0.281 | 0 | 1 |
| June | 370,955 | 0.205 | 0.404 | 0 | 1 |
| July | 370,955 | 0.184 | 0.388 | 0 | 1 |
| Aug-Sep | 370,955 | 0.105 | 0.307 | 0 | 1 |
| Oct-Dec | 370,955 | 0.059 | 0.236 | 0 | 1 |

### Table S3. Descriptive statistics of the sample (IPW method application)

| Variable | Obs. | Mean | Std. dev. | Min | Max |
| --- | --- | --- | --- | --- | --- |
| Exam mark | 222,886 | 25.9 | 3.6 | 18 | 30 |
| Pre-pandemic period | 222,886 | 0.570 | 0.495 | 0 | 1 |
| Covid I | 222,886 | 0.155 | 0.362 | 0 | 1 |
| Covid II | 222,886 | 0.131 | 0.337 | 0 | 1 |
| Covid III | 222,886 | 0.144 | 0.351 | 0 | 1 |
| In-person course | 222,886 | 0.811 | 0.392 | 0 | 1 |
| On-schedule exam | 222,886 | 0.725 | 0.446 | 0 | 1 |
| Female | 222,886 | 0.668 | 0.471 | 0 | 1 |
| Age | 222,886 | 22.7 | 2.7 | 18 | 36 |
| Age squared | 222,886 | 520.4 | 135.3 | 324 | 1296 |
| Born in MO or RE | 222,886 | 0.517 | 0.500 | 0 | 1 |
| Born in ER region | 222,886 | 0.113 | 0.316 | 0 | 1 |
| Born in Italy | 222,886 | 0.310 | 0.463 | 0 | 1 |
| Born abroad and Italian | 222,886 | 0.021 | 0.144 | 0 | 1 |
| Born abroad and non-Italian | 222,886 | 0.038 | 0.192 | 0 | 1 |
| Resident in MO or RE | 222,886 | 0.609 | 0.488 | 0 | 1 |
| Resident in ER region | 222,886 | 0.125 | 0.331 | 0 | 1 |
| Resident out of ER region | 222,886 | 0.266 | 0.442 | 0 | 1 |
| Classical Lyceum | 222,886 | 0.092 | 0.289 | 0 | 1 |
| Scientific Lyceum | 222,886 | 0.305 | 0.460 | 0 | 1 |
| Linguistic Lyceum | 222,886 | 0.115 | 0.319 | 0 | 1 |
| Socio-educational Lyceum | 222,886 | 0.083 | 0.276 | 0 | 1 |
| Other Lyceum | 222,886 | 0.078 | 0.268 | 0 | 1 |
| Commercial school | 222,886 | 0.156 | 0.363 | 0 | 1 |
| Industrial school | 222,886 | 0.036 | 0.186 | 0 | 1 |
| Other technical school | 222,886 | 0.061 | 0.240 | 0 | 1 |
| Professional school | 222,886 | 0.035 | 0.184 | 0 | 1 |
| Other school | 222,886 | 0.024 | 0.152 | 0 | 1 |
| Foreign diploma | 222,886 | 0.016 | 0.125 | 0 | 1 |
| Sophomore or Junior year | 222,886 | 0.652 | 0.476 | 0 | 1 |
| Physiotherapy and Dental hygiene | 222,886 | 0.072 | 0.259 | 0 | 1 |
| Education and Human Sciences | 222,886 | 0.196 | 0.397 | 0 | 1 |
| Communication and Economics | 222,886 | 0.200 | 0.400 | 0 | 1 |
| Economics | 222,886 | 0.213 | 0.409 | 0 | 1 |
| Law | 222,886 | 0.129 | 0.335 | 0 | 1 |
| Medicine and Nursing | 222,886 | 0.191 | 0.393 | 0 | 1 |
| Master student | 222,886 | 0.364 | 0.481 | 0 | 1 |
| Number of exams already passed | 222,886 | 13.95 | 11.06 | 0 | 100 |
| 6 CFU or lower | 222,886 | 0.495 | 0.500 | 0 | 1 |
| 7-9 CFU | 222,886 | 0.362 | 0.480 | 0 | 1 |
| 10 CFU or more | 222,886 | 0.143 | 0.350 | 0 | 1 |
| January | 222,886 | 0.195 | 0.396 | 0 | 1 |
| February | 222,886 | 0.163 | 0.369 | 0 | 1 |
| March-May | 222,886 | 0.102 | 0.303 | 0 | 1 |
| June | 222,886 | 0.206 | 0.404 | 0 | 1 |
| July | 222,886 | 0.178 | 0.382 | 0 | 1 |
| Aug-Sep | 222,886 | 0.098 | 0.297 | 0 | 1 |
| Oct-Dec | 222,886 | 0.059 | 0.235 | 0 | 1 |

### Table S4. IPW first stage estimation

| VARIABLES | Y = Probability of being enrolled in an in-presence course |
| --- | --- |
| Female | -0.030*** |
| Age | -0.036*** |
| Age squared | 0.001*** |
| Born in ER region | -0.001 |
| Born in Italy | 0.008 |
| Born abroad and Italian | -0.004 |
| Born abroad and non-Italian | 0.027** |
| Resident in ER region | -0.066*** |
| Resident out of ER region | -0.024*** |
| Scientific Lyceum | 0.037*** |
| Linguistic Lyceum | -0.020** |
| Socio-educational Lyceum | -0.027*** |
| Other Lyceum | -0.053*** |
| Commercial school | -0.022** |
| Industrial school | 0.006 |
| Other technical school | 0.01 |
| Professional school | -0.022** |
| Other school | -0.002 |
| Foreign diploma | 0.051** |
| Education and Human Sciences | -0.433*** |
| Communication and Economics | 0.044*** |
| Economics | 0.007 |
| Law | -0.135*** |
| Medicine and Nursing | -0.299*** |
| Master student | 0.306*** |
| Sophomore or Junior year | 0.007** |
| Enrollment year = 2018 | -0.030*** |
| Enrollment year = 2019 | -0.043*** |
| Enrollment year = 2020 | -0.005 |
| Observations | 222,886 |

Notes: Standard errors are clustered at student level. * p < 0.1; ** p < 0.05; *** p < 0.01.

### Table S5. Impact of the Covid-19 period on exams’ mark. Students belonging to IPW sample only

|  | Full period | Sub-periods | First year |
| --- | --- | --- | --- |
| Covid | 0.260*** |  | 0.324*** |
| Covid I |  | 0.287*** |  |
| Covid II |  | 0.366*** |  |
| Covid III |  | 0.052 |  |
| Observations | 161,058 | 161,058 | 139,442 |

Notes: Standard errors are clustered at student level. * p < 0.1; ** p < 0.05; *** p < 0.01. While only coefficients of the variables of interest are presented here, all estimates are based on a model specification including covariates listed in Section 4.

### Table S6. Impact of the Covid-19 period on exams’ mark- Heterogeneity by ERC sectors

| ERC sector | Full period | Sub-periods | | | First year |
| --- | --- | --- | --- | --- | --- |
|  | Covid | Covid I | Covid II | Covid III | Covid |
| Life Sciences | 0.302*** | 0.338*** | 0.350*** | 0.197*** | 0.338*** |
| Social Sciences and Humanities | 0.201*** | 0.239*** | 0.279*** | 0.047 | 0.254*** |
| Mathematics, physical sciences, information and communication, engineering, universe and earth sciences | 0.197*** | 0.248*** | 0.210*** | 0.114*** | 0.224*** |
| Total | 0.186*** | 0.233*** | 0.221*** | 0.078*** | 0.224*** |

Notes: Standard errors are clustered at student level. * p < 0.1; ** p < 0.05; *** p < 0.01. While only coefficients of the variables of interest are presented here, all estimates are based on a model specification including covariates listed in Section 4.

### Table S7. Impact of the Covid-19 period on exams’ mark - Heterogeneity by Teachers’ age

| Teacher aged 59 or younger | | | |
| --- | --- | --- | --- |
|  | Full period | Sub-periods | First year |
| Covid | 0.190*** |  | 0.231*** |
| Covid I |  | 0.253*** |  |
| Covid II |  | 0.222*** |  |
| Covid III |  | 0.064*** |  |
| Observations | 304,087 | 304,087 | 261,925 |
| Teacher aged 60 or older | | | |
|  | Full period | Sub-periods | First year |
| Covid | 0.214*** |  | 0.212*** |
| Covid I |  | 0.205*** |  |
| Covid II |  | 0.273*** |  |
| Covid III |  | 0.150*** |  |
| Observations | 66,868 | 66,868 | 57,133 |

Notes: Standard errors are clustered at student level. * p < 0.1; ** p < 0.05; *** p < 0.01. While only coefficients of the variables of interest are presented here, all estimates are based on a model specification including covariates listed in Section 4.

### Table S8. Impact of the Covid-19 on exams’ mark - Interaction with courses’ teaching modality. Heterogeneity by ERC sectors

| Life Sciences | | | | |
| --- | --- | --- | --- | --- |
|  | Base model full period | DID model full period | Base model sub-periods | DID model sub-periods |
| Covid | 0.085 | 0.587* |  |  |
| Covid I |  |  | -0.060 | 0.619 |
| Covid II |  |  | 0.173* | 0.555 |
| Covid III |  |  | 0.135 | 0.942 |
| In-person | 3.047*** | 3.520*** | 3.148*** | 3.723*** |
| In-person*Covid |  | -0.509 |  |  |
| In-person*Covid I |  |  |  | -0.680 |
| In-person*Covid II |  |  |  | -0.385 |
| In-person*Covid III |  |  |  | -0.817 |
| Observations | 10,111 | 10,111 | 10,111 | 10,111 |
| Social Sciences and Humanities | | | | |
|  | Base model full period | DID model full period | Base model sub-periods | DID model sub-periods |
| Covid | 0.334*** | 0.834*** |  |  |
| Covid I |  |  | 0.389*** | 1.119*** |
| Covid II |  |  | 0.434*** | 1.241*** |
| Covid III |  |  | 0.124 | 0.004 |
| In-person | -0.407** | -0.177 | -0.410** | -0.176 |
| In-person*Covid |  | -0.641** |  |  |
| In-person*Covid I |  |  |  | -0.909*** |
| In-person*Covid II |  |  |  | -1.024*** |
| In-person*Covid III |  |  |  | 0.105 |
| Observations | 122,136 | 122,136 | 122,136 | 122,136 |
| Mathematics, physical sciences, information and communication, engineering, universe and earth sciences | | | | |
|  | Base model full period | DID model full period | Base model sub-periods | DID model sub-periods |
| Covid | 0.083* | 1.416*** |  |  |
| Covid I |  |  | 0.040 | 1.125*** |
| Covid II |  |  | 0.343*** | 1.655*** |
| Covid III |  |  | -0.433*** | 1.382*** |
| In-person | 1.271 | 1.742* | 1.329 | 1.789* |
| In-person*Covid |  | -1.355*** |  |  |
| In-person*Covid I |  |  |  | -1.107*** |
| In-person*Covid II |  |  |  | -1.333*** |
| In-person*Covid III |  |  |  | -1.841*** |
| Observations | 28,804 | 28,804 | 28,804 | 28,804 |

Notes: Standard errors are clustered at student level. * p < 0.1; ** p < 0.05; *** p < 0.01. While only coefficients of the variables of interest are presented here, all estimates are based on a model specification including covariates listed in Section 4.

### Table S9. Impact of the Covid-19 on exams’ mark - Interaction with courses’ teaching modality. Heterogeneity by Teachers’ age

|  | Teacher aged 59 or younger | | | | Teacher aged 60 or older | | | |
| --- | --- | --- | --- | --- | --- | --- | --- | --- |
|  | Base model full period | DID model full period | Base model first year | DID model first year | Base model full period | DID model full period | Base model first year | DID model first year |
| Covid | 0.183*** | 0.532* | 0.309*** | 1.219*** | 0.565*** | 1.427*** | 0.560*** | 1.360*** |
| Covid I |  |  |  |  |  |  |  |  |
| Covid II |  |  |  |  |  |  |  |  |
| Covid III |  |  |  |  |  |  |  |  |
| In-person | -0.271 | -0.121 | -0.477*** | -0.189 | -0.642*** | -0.279 | -0.723*** | -0.470* |
| In-person*Covid |  | -0.422 |  | -1.092*** |  | -1.082** |  | -0.995** |
| In-person*Covid I |  |  |  |  |  |  |  |  |
| In-person*Covid II |  |  |  |  |  |  |  |  |
| In-person*Covid III |  |  |  |  |  |  |  |  |
| Observations | 130,132 | 130,132 | 113,037 | 113,037 | 30,926 | 30,926 | 26,405 | 26,405 |

Notes: Standard errors are clustered at student level. * p < 0.1; ** p < 0.05; *** p < 0.01. While only coefficients of the variables of interest are presented here, all estimates are based on a model specification including covariates listed in Section 4.

### Table S10. Impact mobility restrictions on exams’ mark. Heterogeneity by subperiods

|  | Sub-periods | First year |
| --- | --- | --- |
| Covid |  | 0.208*** |
| Covid I | 0.217*** |  |
| Covid II | 0.209*** |  |
| Covid III | 0.074*** |  |
| % red zone last 2 weeks | 0.115*** | 0.137*** |
| Observations | 368,501 | 316,865 |

Notes: Standard errors are clustered at student level. * p < 0.1; ** p < 0.05; *** p < 0.01. While only coefficients of the variables of interest are presented here, all estimates are based on a model specification including covariates listed in Section 4.
